# Assessment of Hyperbolic Heat Transfer Equation in Theoretical Modeling for Radiofrequency Heating Techniques

**DOI:** 10.2174/1874120700802010022

**Published:** 2008-04-10

**Authors:** Juan A López-Molina, Maria J Rivera, Macarena Trujillo, Fernando Burdío, Juan L Lequerica, Fernando Hornero, Enrique J Berjano

**Affiliations:** 1 Applied Mathematics Department, Instituto de Matemática Pura y Aplicada, Universidad Politécnica de Valencia, Valencia, Spain; 2 Department of Surgery, Hospital del Mar, Barcelona, Spain; 3 Cardiac Research Laboratory, Instituto de Biomedicina, Spanish Council for Scientific Research (CSIC), Valencia, Spain; 4 Department of Cardiac Surgery, Hospital General Universitario, Valencia, Spain; 5 Institute for Research and Innovation on Bioengineering, Universidad Politécnica de Valencia,Valencia, Spain

## Abstract

Theoretical modeling is a technique widely used to study the electrical-thermal performance of different surgical procedures based on tissue heating by use of radiofrequency (RF) currents. Most models employ a parabolic heat transfer equation (PHTE) based on Fourier’s theory, which assumes an infinite propagation speed of thermal energy. We recently proposed a one-dimensional model in which the electrical-thermal coupled problem was analytically solved by using a hyperbolic heat transfer equation (HHTE), i.e. by considering a non zero thermal relaxation time. In this study, we particularized this solution to three typical examples of RF heating of biological tissues: heating of the cornea for refractive surgery, cardiac ablation for eliminating arrhythmias, and hepatic ablation for destroying tumors. A comparison was made of the PHTE and HHTE solutions. The differences between their temperature profiles were found to be higher for lower times and shorter distances from the electrode surface. Our results therefore suggest that HHTE should be considered for RF heating of the cornea (which requires very small electrodes and a heating time of 0.6 s), and for rapid ablations in cardiac tissue (less than 30 s).

## INTRODUCTION

Radiofrequency (RF) currents have been employed in many surgical and therapeutic procedures such as the elimination of cardiac arrhythmias and the destruction of tumors. Theoretical modeling has been widely used to investigate and develop new techniques of RF heating (RFH) in biological tissue and to study in depth the electrical and thermal phenomena involved in the process [1]. To date, all theoretical models have employed a heat transfer equation in which the heat conduction term was based on Fourier’s theory, and hence related to heat flux $\left(\vec{q}\right)$ in the following way:

(1) $$\vec{q}\left(\vec{r},t\right)=-k\vec{\nabla }T\left(\vec{r},t\right)$$

where *k* is the thermal conductivity and $T\left(\vec{r},t\right)$ the temperature at point $\vec{r}$ at time *t*. As a result, a parabolic heat transfer equation (PHTE) was employed as the governing equation. Fourier’s theory assumes an infinite thermal energy propagation speed, i.e. any local temperature disturbance causes an instantaneous perturbation in the temperature at each point in the medium [2]. Although this approach might be suitable for most RFH procedures, it has been suggested that under certain conditions (such as very short duration heating employed in RF heating of the cornea [3]), a non-Fourier model should be considered in the form of the hyperbolic heat transfer equation (HHTE), i.e. considering a thermal relaxation time (*τ*) for the tissue ≠0 [2]. As it is known that heat is always found to propagate at a finite speed [4], Cattaneo [5] and Vernotte [6] simultaneously suggested a modified heat flux model in the form:

(2) $$\vec{q}\left(\vec{r},t+\tau \right)=-k\vec{\nabla }T\left(\vec{r},t\right)$$

where *τ* is the thermal relaxation time of the biological tissue. This equation assumes that the effect (heat flux) and the cause (temperature gradient) occur at different times and that the delay between heat flux and temperature gradient is *τ* [4]. The particular case of considering *τ *= 0 obviously corresponds to the Fourier theory.

In order to make a theoretical study of the differences in the temperature profiles obtained from both equations in a general case of RFH, we recently built a one-dimensional model in which the electrical-thermal coupled problem was analytically solved by using both PHTE and HHTE [7]. In this study, in order to precisely quantify the differences between both equations, we particularized that analytical solution to three typical examples of RFH: heating of the cornea for refractive surgery, cardiac ablation for eliminating arrhythmias, and hepatic ablation for destroying tumors.

## ANALYTICAL MODEL

**Fig. (1):**
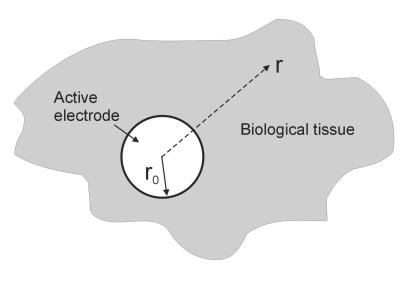
Schematic diagram of the model geometry. **A** spherical electrode (grey circle) of radius *r_0_* is completely imbedded and in close contact with the biological tissue, which has an infinite dimension. As a result, the model presented a radial symmetry, and a one-dimensional approach is possible (dimensional variable is *r*).

Model geometry similar to those proposed by Erez and Shitzer [8] was considered. Briefly, we modeled a *r_0_* radius spherical electrode completely imbedded in and in close contact with the biological tissue (see Fig. **1**), which had an infinite dimension.

The model thus presented radial symmetry and a one-dimensional approach was possible. Regarding the electrical problem, the source term for the RFH modeling (i.e. the Joule heat produced per unit volume of tissue, *Q*(*r,t*)) can be expressed as:

(3) $$Q\left(r,t\right)=\frac{P\cdot r_{0}}{4\cdot \pi \cdot r^{4}}H\left(t\right)$$

where *P* is the total applied power (W), *r*_0_ the electrode radius, and *H(t)* is the Heaviside function. Here we are modeling a protocol of constant power step at t=0. We then considered Özişik and Tzou’s heat transfer model [9]:

(4) $$q\left(r,t\right)+\tau \frac{\partial q\left(r,t\right)}{\partial t}=-k\nabla T\left(r,t\right)$$

which combined with the energy equation:

(5) $$-\nabla q\left(r,t\right)+Q\left(r,t\right)=\rho c\frac{\partial T\left(r,t\right)}{\partial t}$$

where *ρ* is the density and *c* the specific heat, allows to obtain:

(6) $$-\Delta T\left(r,t\right)+\frac{1}{\alpha }\left(\frac{\partial T\left(r,t\right)}{\partial t}+\tau \frac{\partial^{2}T\left(r,t\right)}{\partial^{2}t}\right)=\frac{1}{k}\left(Q\left(r,t\right)+\tau \frac{\partial Q\left(r,t\right)}{\partial t}\right)$$

where *α* is the thermal diffusivity.

Finally, we combined (3) and (6), and we obtained the governing equation (HHTE) for the hyperbolic case:

(7) $$-\alpha \left(\frac{\partial^{2}T\left(r,t\right)}{\partial r^{2}}+\frac{2}{r}\frac{\partial T\left(r,t\right)}{\partial r}\right)+\frac{\partial T\left(r,t\right)}{\partial t}+\tau \frac{\partial T\left(r,t\right)}{\partial t}=\frac{P\alpha r_{0}}{4\pi kr^{4}}\left(H\left(t\right)+\tau \delta \left(t\right)\right)$$

where *δ(t)* is Dirac’s function. To write the boundary condition in *r *= *r*_0_ we adopted a simplification assuming the thermal conductivity of the electrode to be much larger than that of the tissue (i.e. assuming that the boundary condition at the interface between electrode and tissue is mainly governed by the thermal inertia of the electrode) [8]. This condition was also matched to the hyperbolic heat flux. More details on boundary and initial conditions can be found in [7]. The PHTE case was also solved and the solution was equivalent to those found by Erez and Shitzer [8].

## ANALYTICAL SOLUTIONS APPLIED TO RF HEATING CASES

Once the two analytical solutions had been obtained by HHTE and PHTE, they were applied in a theoretical study of temperature distributions in three types of biological tissues: cornea, heart and liver. Electrical and thermal characteristics of these tissues are shown in Table **1** [3,10,11].

There is a lack of experimental data at the present time regarding the tissue thermal relaxation time *τ*(*t*). For cardiac and hepatic tissue we considered a value of *τ* = 16 s (which has been measured in processed meat [12]). Values ranging from 10 to 50 s have been found for non-homogeneous inner structure materials [13], suggesting that non-homogeneity might involve a higher value of *τ*.

Since the cornea has a more homogeneous inner structure than these tissues, we considered the lower value of *τ *= 0.1 s for it. This is obviously a first approximation and experimental data obtained from further studies could give different results. 

Regarding the active electrode, even though a stainless steel active electrode is used in various RFH procedures, in this study we only employed an active electrode made of platinum-iridium, as used in RF cardiac ablation. The characteristics of this electrode were: density 21.5×10^3^ kg/m^3^, specific heat 132 J/kg.K, and thermal conductivity 71 W/m·K [14]. In the three experimental cases, applied power was selected to maintain the maximal temperature in the tissue below 120ºC. Consequently, the power level finally employed was not comparable to those employed clinically, but this was not considered to be important in the context of this study.

## RF HEATING OF THE CORNEA

**Fig. (2):**
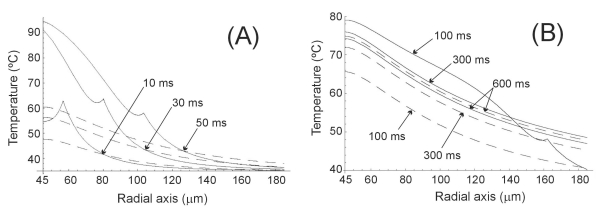
Temperature distributions during RF heating of the cornea along radial axis for different times (from 10 ms to 600 ms) and for the Fourier heat transfer equation (dashed line) and hyperbolic heat transfer equation (solid line). Electrode radius 45 μm. Applied power 30 mW. Thermal relaxation time of the cornea 0.1 s.

Firstly, we considered the case of conductive keratoplasty (CK), in which a small active electrode is inserted into the cornea and a small amount of energy is delivered to the corneal stroma (less than 600 mW for 600 ms) [3]. As the geometry of the active electrode considered in this study (i.e. spherical electrode totally embedded in the tissue) is not the same as the penetrating electrode clinically employed in CK, we chose different parameters: electrode radius 45 μm, and 30 mW constant power applied for 600 ms. The initial temperature was assumed to be 35ºC [3]. Fig. (**2**) shows the temperature distributions along the radial axis for different times from 10 ms to 600 ms for the two heat transfer equations. For shorter times (Fig. **2A**), the HHTE produced temperatures higher than the PHTE. However, this trend became negligible for a longer time t = 600 ms, when both equations gave similar temperatures (see Fig. **2B**). This phenomenon can also be observed in Fig. (**3**), which plots temperature progress at three locations. As can be seen from this figure, at the beginning of heating, the rate of temperature change with HHTE was faster than PHTE and differences became small for longer times. The most remarkable characteristic of the HHTE analytical solution was the presence of cuspidal type singularities. This was materialized as a temperature peak which traveled through the medium at the finite speed of ≈1.25 mm/s (see Fig. **2A**).

## RF CARDIAC ABLATION 

Secondly, we modeled an RF cardiac ablation. In this case, we considered an electrode radius of 1.5 mm, and a power of 3 W for 120 s. The initial temperature was assumed to be 37ºC. Fig. (**4**) shows the temperature distributions along the radial axis for times from 5 s to 120 s for the two heat transfer equations. The differences in both solutions were considerable at the beginning of heating, especially for t ≤ 30 s (see also Fig. **5**).

Once more, in the initial stages HHTE produced higher temperatures than PHTE. Likewise, the presence of cuspidal type singularities from the HHTE were also observed in the case of cardiac ablation as a temperature peak which traveled through the medium at the finite speed of ≈0.1 mm/s (see Fig. **4A**).

## RF ABLATION OF LIVER

Finally, we modeled the RF liver ablation without blood perfusion (typically found in ex vivo experiments). In this case, we considered an electrode radius of 1.5 mm and power of 1 W for 720 s. Initial temperature was assumed to be 37ºC. Fig. (**6**) shows the temperature distributions along the radial axis for five different times (60, 120, 240, 360 and 720 s) for both heat transfer equations. In this third case, which involved longer times, the differences between the equations were almost negligible. Temperature peaks associated with the cuspidal type singularities from the HHTE were only observed for shorter times (see Fig. **7**).

## DISCUSSION

Our indirect objective was to assess the suitability of HHTE for RFH modeling by comparing it to PHTE. For this reason we built a simple geometry model and solved both equations analytically, employing the PHTE model proposed by Erez and Shitzer [8]. We then modified it to include finite thermal propagation speed and calculated the transit-time solution. Finally, we particularized both solutions (HHTE and PHTE) to three RFH examples and compared the results in each case.

**Fig. (3):**
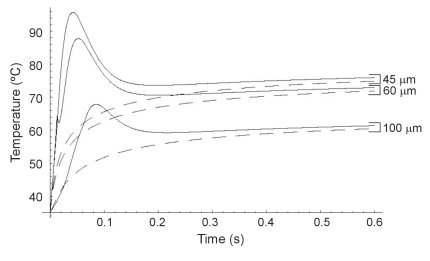
Temperature evolution during RF heating of the cornea (600 ms) at three locations: on the electrode surface, and at 15 μm and 55 μm from the electrode surface. Electrode radius 45 μm. Applied power 30 mW. Thermal relaxation time of the cornea 0.1 s. Fourier heat transfer equation with dashed line, and hyperbolic heat transfer equation with solid line.

**Fig. (4):**
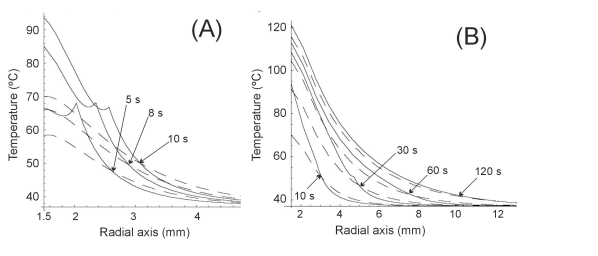
Temperature distributions during RF heating of the heart along radial axis for different times (from 5 s to 120 s) and for the Fourier heat transfer equation (dashed line) and hyperbolic heat transfer equation (solid line). Electrode radius 1.5 mm. Applied power 3 W. Thermal relaxation time of the cardiac tissue 16 s.

**Fig. (5):**
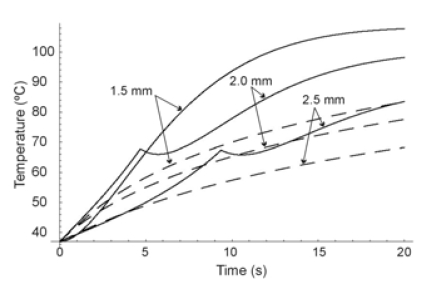
Temperature evolution during RF heating of the heart (first 20 s) at three locations: on the electrode surface, and at 0.5 mm and 1.0 mm from the electrode surface. Electrode radius 1.5 mm. Applied power 3 W. Thermal relaxation time of the cardiac tissue 16 s. Fourier heat transfer equation with dashed line, and hyperbolic heat transfer equation with solid line.

**Fig. (7):**
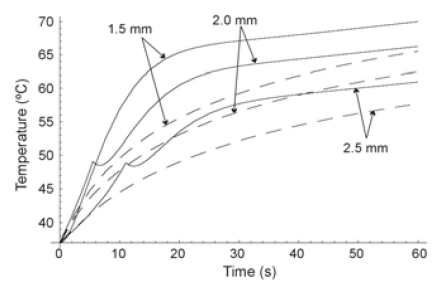
Temperature evolution during RF heating of the liver (first 60 s) at three locations: on the electrode surface, and at 0.5 mm and 1.0 mm from the electrode surface. Electrode radius 1.5 mm. Applied power 3 W. Thermal relaxation time of the hepatic tissue 16 s. Fourier heat transfer equation with dashed line, and hyperbolic heat transfer equation with solid line.

In all three cases we observed similar behavior in the results, which allows us to discuss them jointly. On one hand we observed that at the beginning of the heating (i.e. when the considered time was comparable to or shorter than thermal relaxation time), PHTE provided temperature values lower than those provided by HHTE (see Figs. **2A**, **3**, **4A**, **5** and **7**). This is in agreement with the results obtained by Banerjee *et al. *[15] in a modeling study of laser ablation with pulsed heating. Similar behavior was also partially observed in a modeling study comparing a non-Fourier heat conduction model to the Fourier heat conduction model [16].

On the other hand, the temperature evolution in HHTE was delayed compared to PHTE. In our study this delay was small and only apparent in some plots of Figs. (**3**, **5** and **7**). This phenomenon has also been observed in other modeling studies on tissue heating and can be explained by the fact that when using HHTE, a period of time is needed for the heat to travel to a particular location in the tissue [2]. Once the thermal wave has traveled to a particular point in the tissue, its temperature can even increase above the value predicted by PHTE [2]. We also observed that temperatures computed both from HHTE and PHTE were similar for longer times, as has previously been observed in other modeling studies [2].

Regarding the temperature peaks shown in Figs. (**2A** and **4A**), it can be checked that they traveled at speeds of ≈1.25 mm/s and ≈0.1 mm/s respectively. These correspond approximately with the finite propagation speeds of the thermal wave (*v*) for cornea and cardiac tissue respectively, obtained from the following formula [15]:

(8) $$v=\sqrt{\frac{k}{\rho \cdot c\cdot \tau }}$$

However, this is not surprising since the mathematical solution of HHTE [17] showed the relation between the time and location of the temperature peaks (i.e. $t=\sqrt{\frac{\tau }{\alpha }\left(r-r_{0}\right)}$ ),

which is implicitly contained in equation (8).

To date, different aspects of the hyperbolic heat transfer equation have been proposed and studied. For example, more general versions using two relaxation parameters, one for the heat flux (coincident with our constant τ) and another for the temperature gradient have been considered [18-21]. However, as far as we know, this framework had not been previously applied to model RF heating.

## LIMITATIONS OF THE STUDY 

This modeling study has several limitations which should be pointed out. Firstly, the geometry of our model (spherical electrode totally imbedded in a homogeneous biological tissue with infinite dimension) is very simple compared not only to the realistic geometry of the electrodes employed in clinical practice (semispherical or needle-shape), but also to the heterogeneity of different types of tissue. However, it is necessary to point out that the main purpose of this study was to particularize an analytical solution of HHTE in RFH modeling for three examples of RFH applications. We therefore think that the differences found in this study between both heat transfer equations might be even higher if more realistic electrode geometries were considered (since electrodes with a sharper tip might involve higher temperature gradients). Further modeling studies considering realistic geometries for the active electrodes and obviously based on numerical methods could assess this hypothesis.

Since we employed a constant-power protocol, we did not consider changes in electrical conductivity (σ) of the tissue with temperature (which would be important in constant-voltage or constant-current protocols). We think that the differences found in our study between the two heat transfer equations might be even higher if this thermal dependence were to be taken into account, especially by applying a constant voltage protocol. We support the hypothesis on the well-known effect of thermal feedback in electrosurgical heating [22]. Briefly, when tissue is heated with an electrical protocol based on a constant applied voltage, the tissue’s electrical resistance declines. Then, more current is delivered from the RF generator and tissue temperature increases, thus creating a positive feedback loop.

Finally, future studies should take the tissue damage process into account, using for instance a first order kinetics model rather than a single temperature value [1]. In relation to this, it is necessary to point out that although temperature values from the PHTE and HHTE become similar for longer times, we found considerable differences at the beginning of the heating. For this reason, since a tissue damage function characterizes the total thermal dose throughout heating, its use might offer larger differences between the equations.

Consequently, we think that future studies on numerical RFH modeling, including realistic electrode geometries, changes in electrical conductivity with temperature and a tissue damage function will provide greater differences between the solutions obtained.

In addition, even though under certain circumstances the difference between the temperatures obtained from the two equations were sizable, it is necessary to emphasize that in the simulations we had to assume values for the tissue thermal relaxation times due to the lack of experimental data. In spite of these limitations, we believe that this study is the first step in the development of theoretical models for RFH which include the hyperbolic heat transfer equation and hence are able to provide more accurate RFH modeling. The following section calls attention to some ideas for future work in this area.

## FUTURE RESEARCH

As we have mentioned, the analytical solution obtained for the simple geometry model presented in this study will allow us to validate other numerical models based on, for instance, the finite element method. However, future studies could be conducted using the one-dimensional model presented here. To be more precise, we are interested in modeling not only the heating phase but also the cooling phase (i.e. the period when no electrical power is applied) [8]. We therefore consider that it will be possible to produce more accurate models of some thermal phenomena that occur during RFH, such as thermal latency [23]. This same solution could also allow us study the different results given by PHTE and HHTE when a train of very short pulses is used. For instance, in conductive keratoplasty (CK) a sequence of 4800 brief pulses of 50 μs are applied. By taking into account our first findings (see Fig. **2**), we think that the differences between the two equations could be higher in the case of pulsed power due to the accumulated differences (pulse by pulse) between the temperatures obtained from each heat transfer equation. In fact, the hyperbolic heat transfer equation has been specially employed for heating techniques based on short energy pulses [15,16]. Finally, future experimental work should be conducted to accurately measure the thermal relaxation time (*τ*) of different biological tissues under different conditions. This could mean significant changes in some of the results of our study related to specific RFH cases.

## CONCLUSIONS 

From a mathematical point of view, the HHTE solution has cuspidal type singularities which reflect the wave nature of the thermal problem. At the beginning of the heating (i.e. when the considered time was comparable to or shorter than the thermal relaxation time) PHTE provided temperature values lower than those from HHTE. The HHTE temperature evolution was delayed compared to those from PHTE due to the fact that heat needs a period of time to travel to a particular location inside the tissue.

The differences between PHTE and HHTE temperature profiles were greater for lower times and shorter distances. For this reason, our results suggest that the HHTE should be considered in the case of RF heating of the cornea (heating time 0.6 s), and for short time ablation in cardiac tissue (less than 30 s).

## Figures and Tables

**Fig. (6):**
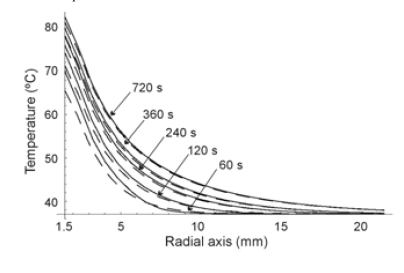
Temperature distributions during RF heating of the hepatic tissue along radial axis for different times (from 60 s to 720 s) and for the Fourier heat transfer equation (dashed line) and hyperbolic heat transfer equation (solid line). Electrode radius 1.5 mm. Applied power 1 W. Thermal relaxation time of the hepatic tissue 16 s.

**Table 1.** Characteristics of the Tissues Used in the Modeling Study (*ρ*: Density; c: Specific Heat; k: Thermal Conductivity)

Tissue	*ρ*(kg/m^3^)	c* (J/kg.K)*	*k*(W/m.K)	Reference
Cornea	1060	3830	0.556	[3]
Heart	1200	3200	0.70	[10]
Liver	1060	3600	0.502	[11]
